# Clove Oil Enhances Fosfomycin Efficacy Against *Escherichia coli* O157:H7 via Biofilm Disruption

**DOI:** 10.3390/biom16060773

**Published:** 2026-05-25

**Authors:** Jing Xu, Zhijin Zhang, Yaxin Zhou, Hongxing Zhang, Zixuan Shang, Guonian Dai, Weiwei Wang, Bing Li, Yubin Bai, Jiyu Zhang

**Affiliations:** 1 Key Laboratory of New Animal Drug Project of Gansu Province, Key Laboratory of Veterinary, Pharmaceutical Development of the Ministry of Agriculture and Rural Affairs, Lanzhou Institute of Husbandry and Pharmaceutical Sciences, Chinese Academy of Agricultural Sciences; New Veterinary Drugs in Gansu Province Engineering Research Center, Key Laboratory of New Veterinary Drugs Engineering, Laboratory Animal Science Data Center of Gansu Province, Lanzhou 730050, China; 2 College of Life Science and Food Engineering, Hebei University of Engineering, Handan 056038, China

**Keywords:** *E. coli O157:H7*, biofilm, *luxS*, QS, *lsrB*, antibacterial synergistic effect

## Abstract

Biofilm formation constitutes a major factor in antibiotic treatment failure, shielding bacteria from drugs and promoting persistence. This study demonstrates that the anti-biofilm action of clove oil enhances the efficacy of fosfomycin against *Escherichia coli O157:H7* (*E. coli* O157). Using a *luxS-eGFP* reporter system, it was found that clove oil inhibited *E. coli* O157 biofilm formation by up to 80% via suppression of the LuxS/AI-2 quorum sensing (QS) system and bacterial motility. Crucially, this disruption was shown to correlate with a strong synergistic effect when combined with fosfomycin in vitro. In a murine peritoneal infection model, the combination therapy demonstrated superior efficacy compared to monotherapy. Specifically, bacterial loads in the liver, spleen, and small intestine were significantly reduced, and histopathological damage was alleviated. Mechanistically, these effects were linked to the downregulation of the QS. These findings indicate that clove oil acts as a potent adjuvant to fosfomycin by disrupting biofilms, offering a promising strategy against systemic infections caused by *E. coli O157*.

## 1. Introduction

The growing problem of antibiotic resistance in recent years is driven not only by horizontal gene transfer and point mutations but also by the complex biofilm structures that bacteria form in specific microenvironments [1]. Biofilms are structured bacterial communities that form as an adaptive strategy. Their enhanced adhesion, heightened pathogenicity, and resistance to antibiotics and host phagocytosis are key reasons for chronic infections and treatment failure [2,3]. Biofilm-associated cells can display antibiotic tolerance that is orders of magnitude higher than that of planktonic cells [4]. According to the National Institutes of Health (NIH), more than 60% of bacterial infections and up to 80% of chronic bacterial infections are associated with biofilm formation [5]. However, standard antibiotic therapies designed for free-floating bacteria often prove ineffective against biofilms, highlighting the urgent need for treatments specifically designed to combat them [6]. As a common Gram-negative opportunistic pathogen, *Escherichia coli* (*E. coli*) serves not only as a model organism in basic research but also forms biofilms that are key to urinary tract infections, catheter-associated illnesses, and food contamination [7,8,9]. *E. coli O157:H7* (*O157*) has attracted significant attention due to its high virulence and notable environmental persistence. As a major foodborne pathogen, the notable biofilm-forming ability of *E. coli O157* is a critical factor for its persistence on farms [10], in processing plants [11], and on kitchen equipment, as well as for its resistance to conventional disinfection procedures [12]. Consequently, selecting *E. coli O157* as a research model allows us not only to target a pathogen of significant public health importance but also to establish an ideal starting point for investigating the common characteristics of biofilms formed by highly pathogenic strains. Furthermore, this research provides a solid theoretical basis and identifies potential therapeutic targets for developing treatments against such prevalent bacterial infections.

The formation of biofilms in *E. coli* is strictly regulated by Quorum Sensing systems (QS). Among these, the LuxS/AI-2 pathway is a hallmark system that plays a central role in regulating motility [13], virulence factor expression, and biofilm development [14,15]. The AI-2 signalling molecule is synthesised by the LuxS protein via the activated methyl cycle and functions as an interspecies signal [16]. Upon reaching a critical concentration, it is taken up by the *Lsr* transport system and activates downstream regulatory networks. Here, we constructed a *luxS-eGFP* reporter to screen plant-derived compounds that perturb LuxS/AI-2 signalling. During the screening of the LuxS/AI-2 system, clove oil exhibited significant signal-interfering activity and was therefore selected as a candidate compound for further investigation. We evaluated the effects of clove oil on quorum sensing gene expression, biofilm formation, and bacterial motility. Additionally, we assessed its antibacterial synergy with fosfomycin sodium both in vitro and in a murine infection model. Clove oil is a volatile extract derived from cloves and is widely used in the food, cosmetics and medical sectors due to its antibacterial, antioxidant, and insecticidal properties [17,18,19]. Although clove oil exhibits broad-spectrum antibacterial and anti-virulence properties, it is unclear whether it functions as a QS inhibitor that specifically disrupts the LuxS/AI-2 pathway to inhibit biofilm formation and function in *E. coli O157*. Furthermore, to assess its potential as an anti-virulence therapy and synergist, we evaluated the therapeutic potential of combining it with the antibiotic fosfomycin sodium. Despite its efficacy against planktonic *E. coli*, fosfomycin sodium exhibits limited efficacy against biofilm-encased cells due to the development of phenotypic tolerance and the protective extracellular matrix.

Therefore, this study aimed to investigate whether clove oil can potentiate fosfomycin efficacy against *E. coli* O157 by disrupting the LuxS/AI-2 mediated regulation. These mechanistic insights were validated in a murine peritoneal infection model to evaluate bacterial clearance and organ protection. Collectively, this work provides a novel, anti-virulence combinatorial strategy to combat refractory biofilm-associated systemic infections.

## 2. Materials and Methods

### 2.1. Materials and Bacteria

The clove oil (containing >85% eugenol), azithromycin (≥98%), polymyxin B (≥98%), cefquinoxime acid (≥98%), streptomycin sulfate (≥98%), chloramphenicol (≥98%), gentamicin (≥98%), fosfomycin sodium (≥98%), and tetracycline (≥98%) to be tested used in this study were purchased from Shanghai Yuanye Biotechnology Co., Ltd. (Shanghai, China). All antibiotics were of analytical grade or higher and used without further purification. Based on its water solubility, it was dissolved in dimethyl sulfoxide (DMSO) to prepare a stock solution with a final concentration of 40 mg/mL, ensuring that the concentration of DMSO in the LB medium did not exceed 1%. Crucially, control groups received an equivalent volume of DMSO without clove oil to serve as vehicle controls.

*E. coli O157:H7* (ATCC 43895) was purchased from the American Typical Culture Collection Center (ATCC, Manassas, VA, USA). Only this single strain was used throughout the study for all in vitro and in vivo experiments. Luria-Bertani (LB, HuanKai Microbial, Guangzhou, China) and Luria-Bertani agar (LA, HuanKai Microbial, Guangzhou, China). The medium is used for the growth of all *E. coli* strains. *Vibrio Harvey BB170* (*V. Harvey Yi BB170*) and *V. Harvey Yi BB152* are gifts from researcher Xiang’an Han (Shanghai Veterinary Research Institute, Chinese Academy of Agricultural Sciences). The culture was carried out in AB medium (the main components in each litre are 0.05 M MgSO_4_, 0.3 M NaCl, and 0.2% vitamin-free Casamino Acids) supplemented with 1 mM L-arginine, 1% phosphate buffer (pH 7.2), and 1% glycerol.

### 2.2. Construction of O157 LuxS-eGFP Vector and Screening Model

Using *luxS*-F and *luxS*-R (Table 1) as primers and the DNA of the *E. coli O157* strain as the template to amplify the *luxS* fragment, the pUC-19 plasmid was selected as the carrier backbone. Using the *luxS* and *eGFP* fragments as templates, the fusion fragment was amplified using the *luxS-eGFP* primer (Table 1). Then, after enzymatic digestion, it was inserted into the pUC-19 vector using T4 DNA ligase to successfully construct the pUC-*LuxS-eGFP* vector. Subsequently, it was transferred to DH5α-activated cells. After activation, the plasmid was extracted, and the vector was transformed by electroporation into the *E. coli O157* competent cell to construct the *luxS-eGFP E. coli O157* tool strain.

### 2.3. LuxS/AI-2 Pathway Screening

The *luxS-eGFP E. coli O157* tool strain was shaken and cultured at 37 °C and 200 rpm until the OD_600_ reached 0.6. Then, IPTG with a final concentration of 1 mM was added, and the culture was incubated for 4 h to induce the fusion expression of *luxS-eGFP*. Using LuxS inhibitors, Furanone C30 (60 μM) as the negative control [20,21], the accuracy of the *luxS-eGFP E. coli O157* strain screening protocol was verified, with Z′ = 0.955, indicating that the model was robust and reliable. When screening drugs, the drugs to be tested were added together with IPTG to the *E. coli O157* bacterial liquid and incubated for 4 h. Then, 200 μL of the bacterial liquid containing various drugs to be tested was taken and added to an opaque 96-well plate. Each group was repeated three times. The luminescence of each bacterial liquid group was detected at an excitation wavelength of 488 nm and an emission wavelength of 509 nm using a multi-functional microplate reader, and this process was repeated three times. Screen out drugs that can inhibit *luxS* fluorescence for the next step of the experiment.

Take *E. coli O157* single colonies in LB broth, activate for 2 generations, adjust to OD_600_ = 1.0, and inoculate them into fresh LB broth at a ratio of 1:100. Co-culture *E. coli O157* with the inhibitor and the drug to be tested for 16 h. After centrifugation at 12,000× *g* for 5 min, the cell-free supernatant was collected using a 0.22 μm filter.

Take a single colony of *V. Harveyi BB170* and inoculate it into AB medium [22]. Activate it for two generations. Inoculate it into AB medium at a ratio of 1:100. Incubate at 30 °C and 160 rpm until OD_600_ = 1.0 approximately. Dilute it into AB medium at a ratio of 1:2500. Mix 20 μL of cell-free supernatant with 180 μL of *V. Harvey Yi BB170* culture and transfer it to a white 96-well plate. Incubate in the dark at 30 °C for 4 h. The amount of bioluminescence was measured using a multi-functional microplate reader. The cell-free supernatant of *V. Harveyi BB152* overnight culture was used as a positive control (*n* = 3).

### 2.4. Metabolic Activity

The metabolic activity of *E. coli* O157 strain was evaluated using the Alamar Blue assay across a concentration range of 25–400 μg/mL of clove oil. After collecting cells from 48-well plates using fresh EP tubes, centrifuging them, washing them with PBS, and resuspending them in 1 mL of PBS, add them to 96-well plates. Add 10 μL of Alamar Blue dye to each well and incubate at 37 °C in the dark for 1 h. A blank control group was established using PBS containing only dyes. The absorbance at 570 nm and 600 nm was detected under a microplate reader, and the metabolic activity was calculated according to the following formula:

$$\mathrm{Metabolic}\;\mathrm{activity}\;(\%)\;=\;\frac{{((E}_{oxi(OD600)}\times T_{OD570})-{(E}_{oxi\left(OD570\right)}\times T_{OD600}))}{{((E}_{red(OD570)}\times B_{OD600})-{(E}_{red(OD600)}\times B_{OD570}))}\times 100\%$$

Eoxi_(OD570)_—extinction coefficient of AB in its oxidized form at 570 nm = 80,586;

Ered_(OD570)—_extinction coefficient of AB in its reduced form at 570 nm = 155,677;

Eoxi_(OD600)—_extinction coefficient of AB in its oxidized form at 600 nm = 117,216;

Ered_(OD600)—_extinction coefficient of AB in its reduced form at 570 nm = 14,652;

B—blank; T—samples.

### 2.5. Biofilms Inhibition

The inhibitory and eradication effects of clove oil on biofilm formation were detected by crystal violet staining (CV). *E. coli* was incubated at 37 °C for 24 h. Subsequently, the bacterial suspension was adjusted to an OD_600_ value of 0.01 and mixed with clove oil at concentrations ranging from 6.25 to 400 μg/mL in a white 96-well plate (Corning Costar^®^ 3599, Corning, Kennebunk, ME, USA). The plate was incubated at 37 °C for another 24 h. Afterward, the plate was rinsed three times with PBS (pH 7.2) to remove non-adherent cells. The biofilm was fixed with methanol and stained with 0.1% (*w*/*v*) CV for 30 min. The unbound dye was removed by rinsing with tap water, and the residual CV in the biofilm was dissolved in 150 mL of 95% ethanol. The absorbance was measured at 595 nanometres. All experiments were performed in three independent replicates.

### 2.6. Biofilms Eradication

*E. coli* was statically cultured at 37 °C for 24 h. The bacterial suspension was adjusted to an OD_600_ value of 0.01, and then 150 μL of the sample was aliquoted into 96-well microplates (Corning Costar^®^ 3599, NY, USA). The samples were left to incubate at 37 °C for 24 h to allow the formation of biofilms. After this, the wells were gently rinsed three times with phosphate-buffered saline (PBS, pH 7.2). Subsequently, pre-formed biofilms were treated with clove oil at concentrations ranging from 50 to 400 μg/mL, and then the samples were cultured at 37 °C for 24 h. After the treatment was completed, the culture medium containing clove oil was carefully removed, and the biofilms were washed three times with sterile PBS. The remaining biofilms were fixed with methanol for 30 min and then stained with 0.1% (*m*/*v*) CV for 10 min. The CV solution associated with the biofilms was washed three times with PBS to remove unbound dyes and then dissolved in 150 mL of 95% ethanol. The absorbance was measured at 595 nm using an enzyme reader. All experiments were performed in three independent replicate experiments.

### 2.7. Scanning Electron Microscopy (SEM) Analysis

Observation of bacterial biofilms by SEM was performed according to previously described methods with minor modifications. Briefly, bacterial suspensions were inoculated into 96-well plates containing cell culture slides and treated with clove oil at concentrations ranging from 100 to 400 μg/mL. The plates were incubated at 37 °C for 24 h to allow biofilm formation. After incubation, the samples were gently washed three times with sterile PBS to remove planktonic bacteria and loosely adherent cells. The cell culture slides were then carefully extracted from the wells and subjected to sequential fixation and gradient ethanol dehydration. Following gold sputter coating, the prepared samples were examined using an FEI Versa 3D scanning electron microscope (Thermo Fisher Scientific, Waltham, MA, USA). All the assays were conducted in three independent replicates (*n* = 3).

### 2.8. Motility Assay

The previous method was adopted to evaluate the effect of clove oil on the motility of *E. coli O157* [23]. In short, the overnight cultured *E. coli O157* suspension was adjusted to OD_600_ = 1.0. A total of 1 μL of the bacterial liquid was inoculated into 0.3% semi-solid LB agar (add 0.3% agar to the LB liquid medium) plates treated with clove oil at concentrations ranging from 100 to 400 μg/mL. After culturing at 37 °C for 24 h, the motility was evaluated by measuring the diameter of the migration halo (compared with the control group). All experiments were set with three independent replicates.

### 2.9. qRT-PCR

The impact of clove oil on the transcription of Quorum Sensing and biofilm regulatory genes in *E. coli O157* was investigated using quantitative real-time PCR (qRT-PCR). *E. coli O157* was co-cultured with clove oil (200 μg/mL) at 37 °C for 24 h, with a control group without clove oil. Total RNA was extracted using the Bacterial RNA Kit (Omega, Norcross, GA, USA). RNA concentration was determined using the NanoDrop OneC spectrophotometer (Thermo Scientific, Waltham, MA, USA). Additionally, RNA was reverse transcribed into cDNA using the PrimeScript™ RT Reagent Kit with gDNA Eraser (TAKARA Corporation, Kusatsu, Japan). qRT-PCR was performed using TB Green^®^ Premix Ex TaqTM II (Tli RNaseH Plus) (TAKARA Corporation, Kusatsu, Japan). The relative changes in gene transcription levels were assessed using the 2^−∆∆Ct^ method. The 16S gene was used as the internal reference gene. The primers used in this study are listed in Table 2. All experiments were set with three independent replicates.

### 2.10. Synergistic Antibacterial Activity

The minimum inhibitory concentrations (MICs) of various antibiotics against *E. coli O157* were determined in accordance with the Clinical and Laboratory Standards Institute (CLSI) document M07, “Methods for Dilution Antimicrobial Susceptibility Tests for Bacteria That Grow Aerobically”. The MIC values were as follows: Azithromycin (0.25 μg/mL), Polymyxin B (1 μg/mL), Cefquinoxime acid (0.0625 μg/mL), Streptomycin Sulfate (2 μg/mL), Chloramphenicol (4 μg/mL), Gentamicin (8 μg/mL), and Tetracycline (0.5 μg/mL). The MIC of fosfomycin Sodium was found to be 8 μg/mL. Based on previous methods [24], we evaluate the synergistic antibacterial effect of clove oil. Briefly, after overnight culture, the bacteria were diluted to an OD_600_ of 0.01. The diluted bacterial solution was mixed with antibiotics (1/2 MIC, 1/4 MIC, 1/8 MIC) with or without clove oil (400 μg/mL). The metabolic activity of the mixed suspension was analysed using the Alamar Blue assay after incubation at 37 °C for 16 to 18 h. The tests were performed in triplicate (*n* = 3).

### 2.11. Evaluation of the Synergistic Antibacterial Effect of Combined Antibiotics In Vivo

Female 6w C57BL/6J mice weighing 18–22 g were purchased from the Lanzhou Veterinary Research Institute of the Chinese Academy of Agricultural Sciences. The animal experiments related to this trial were conducted in accordance with the regulations on the management and use of experimental animals of the Lanzhou Institute of Husbandry and Pharmaceutical Sciences of the Chinese Academy of Agricultural Sciences and were approved by the Ethics Committee. After a 3-day acclimatization period, the mice were randomly divided into four groups: the control group, the fosfomycin sodium group, the clove oil group, and the clove oil combined with fosfomycin sodium group, with 12 mice in each group. *E. coli O157* was cultured overnight and passaged once. The bacterial suspension was adjusted to 0.5 McFarland standard (equivalent to 1.0 × 10^8^ CFU/mL) using a McFarland turbidimeter, and then further diluted to a final concentration of 1.5 × 10^9^ CFU/mL. On the first day of the experiment, the bacterial suspension was intraperitoneally injected at a dose of 0.2 mL/10 g. To evaluate therapeutic efficacy, bacterial inoculation and drug administration were performed simultaneously via the same intraperitoneal route. One hour after infection, clove oil (1 mg/mL, dissolved in 0.1% Tween 80) were intraperitoneally injected; the combination group received both fosfomycin sodium (2.5 mg/mL) and clove oil (1 mg/mL) simultaneously. The control group received an equal volume of 0.1% Tween 80. The mice were observed for seven days with free access to food and water. After seven days, mice were euthanised via cervical dislocation following respiratory anaesthesia with isoflurane (RWD, Shenzhen, China). The required tissues were collected and frozen or fixed for preservation.

### 2.12. Immunofluorescence, Hematoxylin, and Eosin

The intestinal tissue was fixed in 4% paraformaldehyde (Servicebio, Wuhan, China), dehydrated, and washed with ethanol and xylene. The transparent tissue blocks were immersed in molten paraffin, and after the paraffin solidified, they were sectioned into 5-8-micrometre-thick slices. Prior to primary antibody incubation, the sections were blocked with serum for 30 min to reduce background staining. After washing three times, the sections were incubated overnight at 4 °C with primary antibodies (ZO-1 and occludin), followed by incubation with the secondary antibody in the dark at room temperature for 1 h. Finally, the sections were counterstained with DAPI and mounted.

Tissues were fixed in 4% paraformaldehyde (Servicebio, China), dehydrated, and washed with ethanol and xylene. The clear tissue blocks were immersed in melted paraffin and cut into 5–8 μm slices after the paraffin had solidified [25].

### 2.13. Statistical Analysis

Statistical analysis and visualisation were conducted with GraphPad Prism (version 10.0, GraphPad Software, San Diego, CA, USA). All measurement data were presented as mean ± standard error of the mean (SEM). Comparisons between the two groups were performed using multiple *t*-tests, while multiple comparisons were conducted using non-parametric one-way ANOVA. The significance threshold was established at α = 0.05, with a difference being statistically significant when the *p*-value < 0.05.

## 3. Results

### 3.1. Effects of Clove Oil on the LuxS/AI-2 QS Pathway

**Figure 1:**
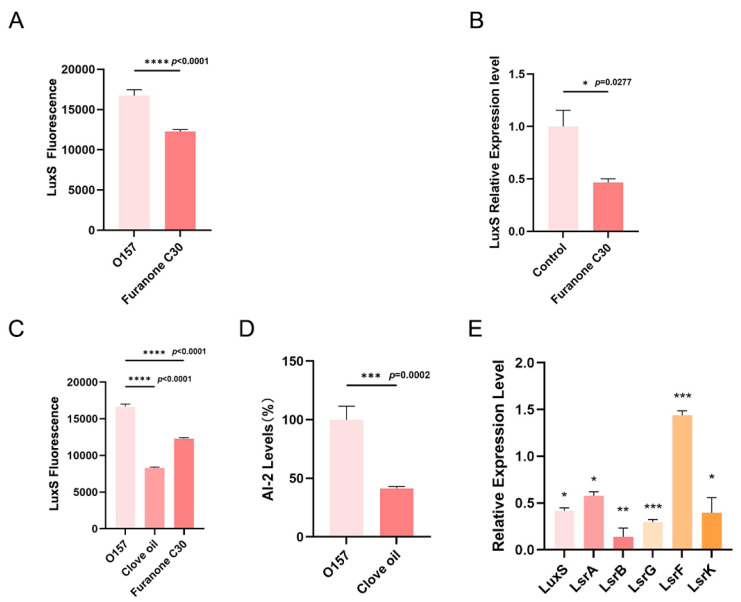
Functional characterization of clove oil using a *luxS-eGFP* reporter. (**A**) Fluorescence intensity of the *luxS-eGFP* reporter strain after treatment with the furanone C30. (**B**) Relative mRNA expression level of the luxS gene in the reporter strain. (**C**) Inhibition of *luxS-eGFP* fluorescence by clove oil. (**D**) The level of AI-2 after treatment with clove oil. (**E**) The mRNA expression levels of genes associated with the *E. coli* QS system (*luxS*, *lsrA*, *lsrB*, *lsrG*, *lsrF*, *lsrK*,) after treatment with the clove oil. (*n* = 3 per group; Data represent means ± SEM; * *p* < 0.05, ** *p* < 0.01, *** *p* < 0.001, **** *p* < 0.0001).

To identify potential QS inhibitors targeting the LuxS/AI-2 system in *E. coli* O157:H7, a luxS-eGFP reporter system was constructed. As shown in Figure 1A, the furanone C30 significantly suppressed both the fluorescence intensity and the transcriptional expression of the *luxS* gene (Figure 1B), confirming the system’s specific responsiveness to LuxS/AI-2 pathway inhibition. This confirmed that the reporter system reliably responds to perturbations in the LuxS/AI-2 QS pathway. Following successful validation, the system was employed to investigate the anti-QS potential of clove oil. Clove oil treatment markedly inhibited the fluorescence intensity of the *luxS-eGFP* reporter strain (Figure 1C) and substantially reduced the extracellular level of the AI-2 signal molecule (Figure 1D). Furthermore, qPCR analysis showed that clove oil downregulated the mRNA expression of key genes in the *E. coli* QS network, including those involved in AI-2 signal processing (Figure 1E).

### 3.2. Effects of Clove Oil on Metabolic Activity and Motility in E. coli O157

**Figure 2:**
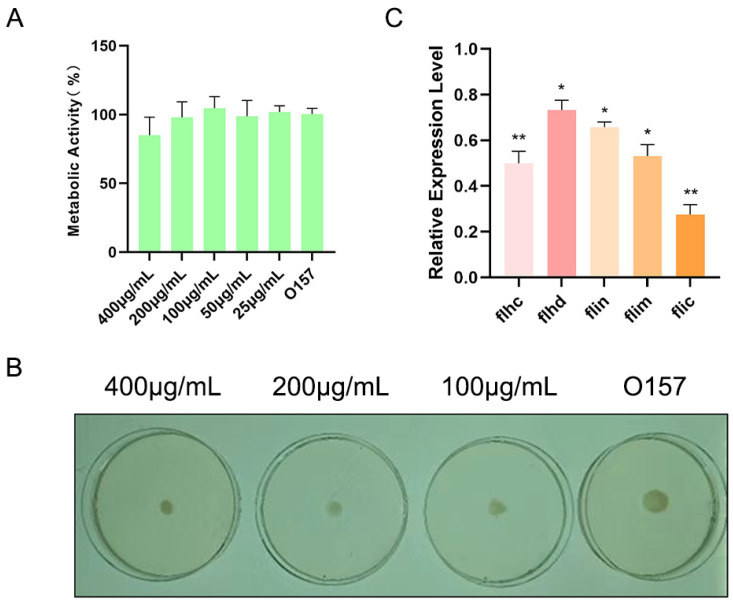
Clove oil inhibits motility and associated metabolic processes in *E. coli O157*. (**A**) The effect of clove oil on the metabolic activity of *E. coli O157*. (**B**) Halo diameter-based quantification of the concentration-dependent inhibition of *E. coli* motility by clove oil. (**C**) Effect of clove oil on mRNA levels of motility genes (*flhC*, *flhD*, *fliN*, *fliM*, *fliC*) in *E. coli*. (*n* = 3 per group; Data represent means ± SEM; * *p* < 0.05, ** *p* < 0.01).

The effect of clove oil on growth and metabolic activity of *E. coli O157* was assessed using the Alamar Blue assay. Metabolic activity was not significantly altered by clove oil within the tested concentration range of 25 to 400 μg/mL (Figure 2A). Subsequently, the motility of *E. coli O157* was examined in the presence of clove oil. Treatment with clove oil resulted in a concentration-dependent inhibition of bacterial motility, as evidenced by a reduction in the migration halo diameter (Figure 2B). Concurrent qRT-PCR analysis revealed that clove oil significantly altered the mRNA expression levels of key motility-associated genes in the strain (Figure 2C).

### 3.3. Effect of Clove Oil on E. coli O157 Biofilm Formation

**Figure 3:**
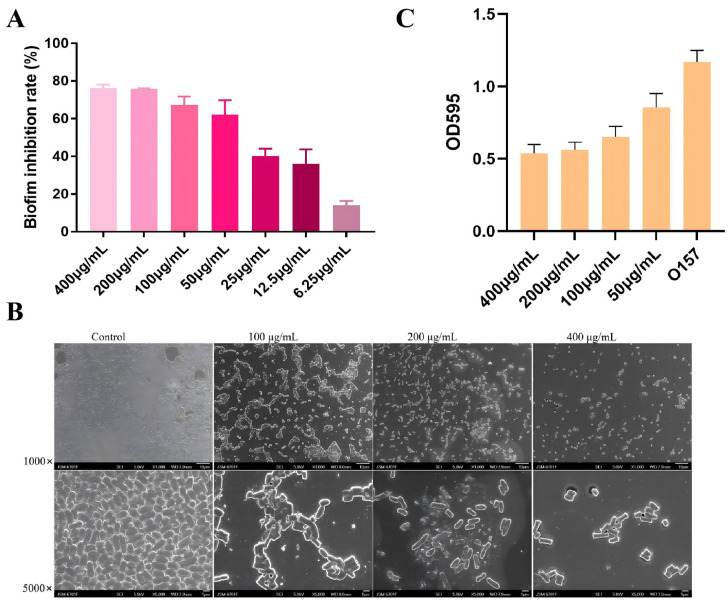
Inhibitory effects of clove oil on *E. coli O157* biofilm formation. (**A**) Inhibition rates of *E. coli* biofilm formation at different clove oil concentrations. (**B**) Scanning electron micrographs of *E. coli O157* treated with different concentrations of clove oil. (**C**) Quantification of *E. coli O157* biofilm formation after treatment with clove oil. (*n* = 3 per group; Data represent means ± SEM).

The effect of clove oil on *E. coli O157* biofilm formation was evaluated by quantifying biofilm biomass using the crystal violet staining assay. As shown in Figure 3A, the inhibitory effect was positively correlated with the concentration of clove oil. The inhibition rate gradually decreased with decreasing concentration (200, 100, 50, 25, 12.5, and 6.25 μg/mL), with the highest concentration (400 μg/mL) exhibiting the most significant effect. At concentrations above 50 μg/mL, clove oil inhibited biofilm formation by more than 60%, reaching nearly 80% inhibition at 400 μg/mL. Furthermore, the morphological and aggregative state of *E. coli O157* after clove oil treatment was observed by scanning electron microscopy (SEM). Figure 3B shows that the untreated control exhibited robust bacterial aggregation and a typical biofilm matrix structure. Treatment with 100 μg/mL and 200 μg/mL clove oil reduced both the degree of aggregation and the amount of matrix. At 400 μg/mL, the number of observable bacteria was substantially diminished, with minimal cell aggregation or biofilm architecture apparent. Consistent with the SEM observations, the total biofilm biomass was quantified after treatment with various clove oil concentrations. As shown in Figure 3C, the amount of biofilm formation, determined by OD_595_ measurement, correlated well with the inhibition rate results. The control group yielded the highest biofilm biomass. Clove oil treatment significantly reduced biomass, as evidenced by the lower biomass at 200 µg/mL compared to 100 and 50 µg/mL. Notably, the effect appeared to reach a plateau at 200 µg/mL, suggesting that 400 µg/mL may not yield a substantially greater inhibitory effect.

### 3.4. Effects of the Clove Oil–Antibiotic Combination on Antibacterial Potentiation

**Figure 4:**
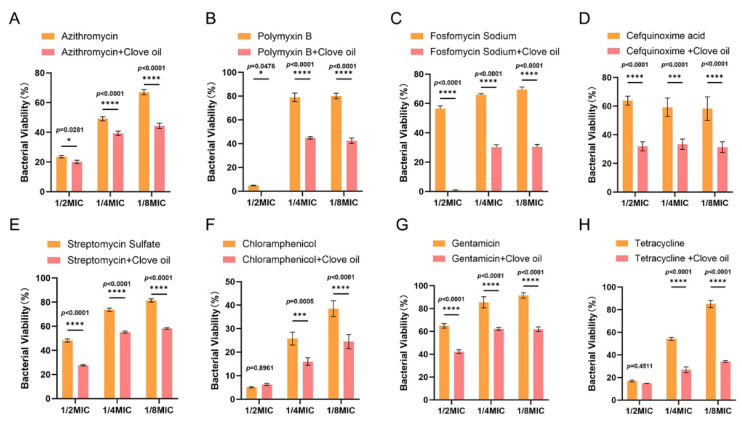
Effects of antibiotic–clove oil combination on bacterial viability. Bar graphs (**A**–**H**) show the viability of *E. coli O157* treated with eight common antibiotics (azithromycin, polymyxin B, fosfomycin sodium, cefquinoxime acid, streptomycin, chloramphenicol, gentamicin, and tetracycline) at sub-inhibitory concentrations (1/2×, 1/4×, and 1/8× MIC), either alone or in combination with a fixed concentration of clove oil. (*n* = 3 per group; Data represent means ± SEM; * *p* < 0.05, *** *p* < 0.001, **** *p* < 0.0001).

Subsequently, experiments were performed to assess the combined effect of clove oil with various antibiotics against *E. coli*. This study demonstrated that the combination of clove oil with each of the eight antibiotics significantly enhanced antibacterial activity at sub-inhibitory concentrations (Figure 4A–H). At all three concentrations tested (1/2, 1/4, and 1/8 MIC), the combination treatment demonstrated superior antibacterial activity compared to the antibiotic alone. At the 1/2 MIC concentration, the combination of clove oil with polymyxin B or fosfomycin reduced bacterial viability to a minimal level (≤5%), demonstrating a potent synergistic bactericidal effect compared to their respective monotherapies. And the effect was particularly notable in the case of fosfomycin sodium. Additionally, combinations of clove oil with cefquinoxime acid, streptomycin, and gentamicin also significantly reduced bacterial viability. In contrast, combinations of clove oil with chloramphenicol or tetracycline did not exhibit clear synergistic effects at the 1/2 MIC. At the 1/4 MIC concentration, the potentiation effect of clove oil remained widespread across the tested antibiotics. The enhancement was particularly pronounced for fosfomycin, with the combination reducing bacterial viability by half compared to its monotherapy. Combinations with polymyxin B, cefquinoxime acid, azithromycin, and gentamicin also exhibited strong synergistic effects, which were lower than those achieved by the corresponding antibiotics alone. In contrast, clove oil combined with chloramphenicol or tetracycline reduced bacterial viability to approximately 20%. At the lowest tested concentration, the potentiation effect of clove oil remained discernible. Notably, the synergy between clove oil and azithromycin remained the most stable, with the combination treatment maintaining a clear advantage in survival rate compared to the antibiotic alone. Taken together, clove oil demonstrated consistent enhancement of the antibacterial activity of azithromycin at all three sub-inhibitory concentrations tested and exhibited marked synergy at different concentrations.

### 3.5. The Synergistic Antibacterial Efficacy of Clove Oil with Fosfomycin Sodium In Vivo

**Figure 5:**
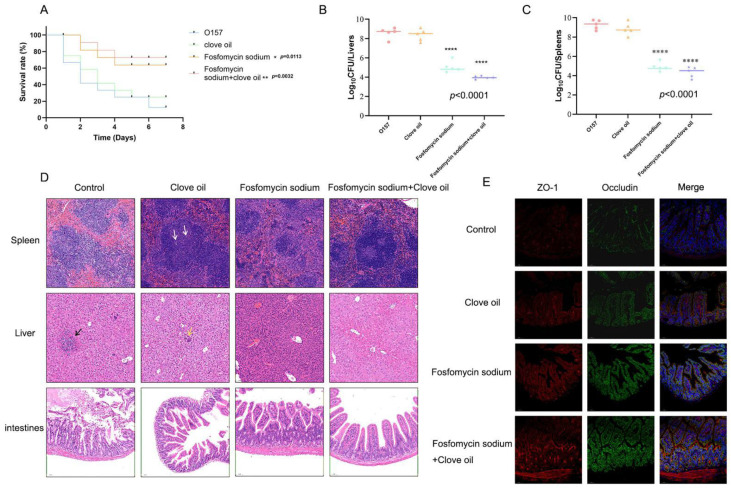
The therapeutic efficacy of clove oil in combination with fosfomycin sodium against *E. coli O157* infection in a murine model. (**A**) Survival of mice following *E. coli O157* infection under different treatments. Mice (*n* = 12 per group) were treated with vehicle (*E. coli O157*), clove oil alone, fosfomycin sodium alone, or their combination. (* *p* < 0.05, ** *p* < 0.01). (**B**) Bacterial loads in the livers of *E. coli O157*-infected mice after different treatments. Mice were treated with vehicle (*E. coli O157*), clove oil alone, fosfomycin sodium alone, or their combination. (*n* = 3 per group; Data represent means ± SEM; **** *p* < 0.0001). (**C**) Bacterial loads in the spleens of *E. coli O157* -infected mice after different treatments. Mice were treated with vehicle (*E. coli O157*), clove oil alone, fosfomycin sodium alone, or their combination. (*n* = 3 per group; Data represent means ± SEM; **** *p* < 0.0001). (**D**) Histopathological analysis of spleen, liver, and intestine from *E. coli O157*-infected mice after treatment. White arrow: granulocyte infiltration; Black arrow: focal necrosis of hepatocytes, fragmentation of nuclei, eosinophilic red-stained cytoplasm, and few lymphocytes; Yellow arrow: granulocyte infiltration. (**E**) Immunofluorescence staining of intestinal tight junction proteins in *E. coli O157*-infected mice. Mice were treated with vehicle (*E. coli O157*), clove oil alone, fosfomycin sodium alone, or their combination.

Consequently, the fosfomycin sodium–clove oil combination was progressed to a murine infection model for further investigation, given the consistent synergy observed in vitro. The combined therapeutic effect of clove oil and fosfomycin sodium was evaluated in a mouse model of intraperitoneal *E. coli O157* infection by assessing pathological changes in the liver, spleen, and ileum tissue. As shown in Figure 5A, the combination therapy of fosfomycin sodium and clove oil resulted in the highest survival rate, significantly outperforming both the control and clove oil group. Fosfomycin sodium monotherapy also provided a substantial survival benefit compared to the control.

The study showed that treatment with clove oil alone did not reduce the bacterial burden (Figure 5B,C). In contrast, monotherapy with fosfomycin sodium significantly reduced the bacterial load, lowering the mean CFU to approximately 5-6 Log_10_ CFU in both the liver and spleen. Notably, the combination of fosfomycin sodium and clove oil proved to be the most effective treatment. It further and significantly decreased the bacterial loads to the lowest observed level (approximately 4 Log_10_ CFU) in the liver (Figure 5B) and spleen (Figure 5C), demonstrating efficacy superior to that of fosfomycin sodium alone.

Histopathological examination of the liver, spleen, and intestines revealed organ-specific effects of the treatments (Figure 5D). In the infection model control group, extensive and ill-defined haemorrhagic necrotic foci (reddish-purple) were observed in the spleen, accompanied by severe disruption of the normal splenic corpuscle architecture, indicating substantial infection-induced splenic inflammation and injury. Treatment with clove oil alone moderately reduced inflammatory cell infiltration and hepatocyte damage, showing a better outcome than the control. In contrast, fosfomycin sodium monotherapy markedly diminished haemorrhagic necrosis and considerably restored splenic structure. Most notably, the combination of fosfomycin sodium and clove oil produced the most pronounced therapeutic effect: haemorrhagic necrotic lesions were nearly absent, splenic corpuscles appeared clearly defined with only mild residual inflammation, and the overall morphology closely resembled that of normal spleen tissue. Histopathological examination of liver tissues revealed distinct responses across treatment groups. The control group exhibited disordered hepatic cord architecture with extensive inflammatory cell infiltration and punctate necrosis around central veins and portal areas, confirming severe infection-induced hepatitis. Clove oil monotherapy alleviated inflammatory infiltration and modestly reduced hepatocellular injury relative to control levels. Fosfomycin sodium alone effectively suppressed inflammation and promoted restoration of regular hepatocyte arrangement, indicating notable hepatoprotective activity. The most pronounced recovery was observed in the combination group, where fosfomycin sodium plus clove oil substantially reduced inflammatory infiltration, restored normal hepatic cord organization, and markedly ameliorated tissue damage, representing the most favourable outcome among all treatments. In the mouse model of *E. coli O157* infection, significant damage to the small intestinal mucosal structure was observed, manifested specifically as villous atrophy and disruption of the tissue structure. Treatment with clove oil alone did not adversely affect intestinal morphology, whereas the administration of fosfomycin sodium alone partially improved the infection-induced damage to mucosal morphology. It is worth noting that when clove oil was administered in combination with sodium fosfomycin, it further significantly alleviated the intestinal mucosal tissue damage associated with sodium fosfomycin and promoted the recovery of villus morphology.

Immunofluorescence staining of intestinal tight junction proteins showed that *E. coli O157* severely disrupted their continuous distribution, indicating compromised barrier function. And administration of clove oil, fosfomycin sodium, or the combination therapy resulted in increased expression of the tight junction proteins ZO-1 and occludin in intestinal tissues compared to the control group. Crucially, the combination therapy of fosfomycin sodium and clove oil effectively preserved the normal, continuous expression pattern of both proteins, demonstrating protection of intestinal barrier integrity.

## 4. Discussion

The ability of bacteria to form biofilms represents a key clinical challenge, as this mode of growth confers enhanced resistance to antibiotics [26]. Therefore, developing agents aimed at disrupting the biofilm matrix to enhance antibiotic action is crucial. Quorum sensing (QS) serves as a pivotal bacterial communication system for regulating diverse behaviours, with the LuxS/AI-2 pathway playing a central role in biofilm formation. Pressing this QS signalling system can reduce bacterial movement, resulting in a considerable inhibition of biofilm development [27].

After validation of the reporter system with the known QS inhibitor furanone C30, clove oil was found to inhibit the fluorescence of the reporter, and it downregulates AI-2 levels. LuxS is the key enzyme for producing the universal signal molecule AI-2 [28], and its downregulation was strongly supported by follow-up data. Furthermore, qPCR analysis confirmed that clove oil not only decreased the mRNA level of *luxS* but also changed other key genes (*IsrA*, *IsrB*, *Isrf*, *IsrK*, *csgD*) within the QS network. In the biofilm formation pathway of *E. coli,* particularly in the LuxS/AI-2 QS system, the genes *lsrA*, *lsrB* and *lsrK*, function in a sequential and dependent manner to translate the intercellular chemical signal into the production of structural biofilm components [29]. When bacteria sense sufficient AI-2 molecular signals, the proteins LsrA and LsrB form an ABC transporter complex responsible for the active import of the extracellular quorum-sensing signal molecule AI-2 into the bacterial cytoplasm [30]. This is the critical first step in perceiving population density. And once internalised, AI-2 must be activated to exert its regulatory function, a key step mediated by the kinase *LsrK* [31]. Following its import, AI-2 is then phosphorylated by the kinase *LsrK*. This phosphorylation transforms the intercellular signal into an intracellular effector, which binds to and inactivates the transcriptional repressor LsrR. The consequent derepression of the lsroperon creates a positive feedback loop, driving the rapid uptake and processing of extracellular AI-2 [32]. The function inhibition of LsrR by AI-2-P leads to the activation of multiple gene systems. A key target activated through this signaling cascade is the *csgD* gene, a master transcriptional regulator that directly activates the operons responsible for producing the major biofilm matrix components, thereby enabling extracellular matrix synthesis and structured biofilm formation [33]. This study demonstrated that clove oil effectively suppresses the expression of key genes in the LuxS/AI-2 QS pathway (*luxS*, *lsrA*, *lsrB*, *lsrK*).

Furthermore, previous studies have shown that clove oil exhibits significant anti-Quorum Sensing (QS) activity in standard inhibitory models. It concentration-dependently suppresses QS-regulated violacein production in Chromobacterium violaceum and reduces the swarming motility of Pseudomonas aeruginosa by up to 78% [17]. By targeting the Quorum Sensing system, clove oil also interferes with the cell–cell communication in Pseudomonas aeruginosa, which in turn suppresses its ability to form biofilms [34]. The findings revealed the specific molecular mechanism underlying the anti-biofilm action of clove oil by interfering with the synthesis, uptake, and transduction of the AI-2 signal. Clove oil blocks this signalling pathway, thereby inhibiting the production of the core transcriptional regulator *csgD*, which controls biofilm matrix synthesis. Consequently, the ability of bacteria to form biofilms is globally suppressed. Furthermore, clove oil also promoted the expression of the *lsrF* gene, which encodes the enzyme responsible for degrading the intracellular active signal molecule AI-2 [32]. This suggests a synergistic, dual-action inhibitory mechanism: while suppressing signal synthesis, uptake, and transduction, it simultaneously accelerates the clearance of existing signal molecules. This dual effect can lead to a more rapid and thorough quenching of the AI-2 signalling pathway, thereby collectively attenuating biofilm formation.

Bacterial motility serves as a key regulatory factor during the four stages of biofilm formation, governing both bacterial aggregation and dispersal [32]. *E. coli* biofilm development comprises four main stages: initial attachment, development, maturation, and dispersal [35]. Bacterial motility is a key regulatory mechanism during biofilm formation, and inducing the dispersal of mature biofilms can prevent biofilm formation by *Staphylococcus aureus* and *E. coli* [36]. The results of motility-related gene (*flhC*, *flhD*, *flhN*, *fliM*, and *fliC*) expression analysis showed that clove oil significantly suppressed their mRNA levels. Notably, the mRNA levels of *flh*C and *flhD*, encoding the master transcriptional regulators of the flagellar system [37], were downregulated to approximately 50% and 70% of the control levels, respectively. This suppression of the *flhD* operon, a key multifunctional regulator, likely initiates a cascade effect on downstream flagellar genes. And the *flhC* gene is crucial for bacterial motility, pilus formation, and adhesion processes and is also involved in biofilm formation [38]. Correspondingly, the expression of key structural genes responsible for flagellar assembly (*fliN*), motor switching (*fliM*), and filament formation (*fliC*) was significantly reduced [35], with expression levels even further decreased than those of the master regulators. As a class II flagellar gene, *fliM* is expressed early during flagellar biosynthesis [39]. In contrast, *fliC* (class III flagellar gene) encodes one of the most abundant bacterial proteins and can participate in signal transduction pathways involved in biofilm formation [40,41]. These results indicate that clove oil broadly inhibits the expression of flagellar-related genes at multiple hierarchical levels, thereby attenuating bacterial motility transcriptionally. This finding provides molecular evidence supporting the mechanism by which clove oil inhibits biofilm formation: by interfering with the transcription of flagellar-associated genes, clove oil likely restricts initial bacterial adhesion and aggregation on substrates, thereby disrupting a critical early step in biofilm development. Correspondingly, the halo assay clearly demonstrated a concentration-dependent inhibition of *E. coli* motility by clove oil. As the clove oil concentration increased from 100 to 400 μg/mL, the diameter of the bacterial migration zone decreased correspondingly, indicating a concentration-dependent suppression of motility. This phenotypic evidence strongly aligns with the transcriptional downregulation of key flagellar genes; clove oil compromises motility by suppressing the flagellar system at the genetic level, which is functionally manifested as impaired migration. However, this motility inhibition occurred without impairing overall metabolic activity. This indicates that the biofilm inhibition is primarily mediated by the specific suppression of motility-related functions, not by a general compromise of bacterial viability.

Subsequently, this study systematically evaluated the inhibitory effect of clove oil on biofilm formation by *E. coli O157* using crystal violet quantification and SEM. At concentrations above 50 μg/mL, clove oil suppressed biofilm formation by more than 60%, reaching nearly 80% inhibition at 400 μg/mL. This dose–response relationship aligns with the previously observed suppression of motility, further supporting the key mechanism of inhibiting initial bacterial attachment. SEM imaging visually revealed the morphological impact of clove oil. As the concentration increased, bacterial aggregates and extracellular matrix structures became progressively sparser, with typical biofilm architecture nearly absent at 400 μg/mL. This structural disruption corresponds closely with the quantitative reduction in biofilm biomass. Notably, the reduction in biofilm biomass showed a plateau effect between 200 and 400 μg/mL, suggesting that the inhibitory activity may approach saturation at higher concentrations. This observation provides important insight for determining the minimal effective biofilm-inhibitory concentration of clove oil. Moreover, clove oil also significantly inhibits biofilm formation in Candida albicans by suppressing adhesion, hyphal development, and interfering with the expression of key MAPK pathway-related genes such as CDC42 and CEK1 [42]. In addition, numerous studies have demonstrated that clove oil significantly inhibits the biofilm formation of Candida albicans [43]. Beyond its efficacy against common pathogens, clove oil also exhibits anti-biofilm activity against industrial contaminants such as the acidophilic thermophile Alicyclobacillus acidoterrestris [44]. Studies have shown that 0.05% (*v*/*v*) clove oil reduces biofilm formation by 25.1–65.0% on surfaces such as glass and polyvinyl chloride, while also altering biofilm architecture, promoting the release of extracellular polymeric substances, and facilitating bacterial detachment. These findings further support the broad-spectrum anti-biofilm potential of clove oil, which extends not only to medically relevant bacteria like *E. coli* but also to spoilage microorganisms in food-processing environments. Furthermore, studies have demonstrated that clove oil can significantly interfere with the QS of *Pseudomonas aeruginosa*, thereby suppressing the production of QS-regulated virulence factors such as proteases and pyocyanin and reducing biofilm formation in a concentration-dependent manner [34]. Additionally, protective effects have also been observed in a nematode infection model [34]. These findings indicate that clove oil not only directly inhibits *E. coli O157* biofilm formation but also attenuates pathogenicity and biofilm development in a broader range of pathogens by disrupting the conserved QS regulatory mechanism. This further highlights the multi-target and cross-species anti-biofilm potential of clove oil. It is important to note that while fosfomycin sodium is a highly effective first-line therapy for acute urinary tract infections caused by planktonic *E. coli*, its efficacy against mature biofilms is often limited [45]. The observed bacterial viability of approximately 60% for fosfomycin alone in our in vitro biofilm model (Figure 4) aligns with its known phenotype of biofilm tolerance. This underscores the clinical necessity for adjuvants like clove oil, which target biofilm-specific resistance mechanisms (such as QS and matrix production) to restore the susceptibility of the bacteria to the antibiotic.

Sub-inhibitory concentrations of clove oil significantly enhanced the efficacy of multiple antibiotics against *E. coli*, showing potent synergy with fosfomycin sodium that reduced viability to ≤5% at 1/2 MIC. This consistent synergistic effect across concentrations is likely due to clove oil’s ability to disrupt membrane integrity, inhibit biofilm formation, and interfere with Quorum Sensing. These in vitro findings strongly support further in vivo evaluation of the fosfomycin–clove oil combination as a promising strategy to combat resistant infections, laying a clear foundation for its testing in murine infection models. As shown in Figure 5, while clove oil alone failed to significantly reduce bacterial loads in organs, its combination with fosfomycin sodium demonstrated a remarkable synergistic antibacterial and tissue-protective effect. The observed synergistic effect likely arises from the complementary mechanisms: fosfomycin sodium acts primarily through the inhibition of cell wall synthesis [46], whereas sub-inhibitory clove oil complements this action by suppressing biofilm formation and interfering with Quorum Sensing, thereby impairing bacterial colonisation, survival, and immune evasion. This potentially enhances the penetration and efficacy of fosfomycin sodium. Notably, the combination therapy group exhibited the lowest bacterial burdens (~4 log_10_ CFU) and the mildest haemorrhagic necrotic lesions and inflammatory infiltration in the spleen and liver, indicating a superior capacity to control systemic infection. More importantly, the combination therapy showed advantages over monotherapies in intestinal mucosal repair and barrier protection. Histopathology revealed that the combined treatment most effectively alleviated infection-induced villous atrophy and structural disruption in the small intestine. Immunofluorescence results further confirmed that the combination most effectively preserved the normal, continuous distribution of tight junction proteins (ZO-1 and occludin). Compromised intestinal barrier integrity is a key step facilitating bacterial translocation and systemic inflammation [47]. The anti-inflammatory and antioxidant properties of clove oil likely played a crucial role [45,46]. Thus, the complementary actions of fosfomycin sodium and clove oil achieve combined bactericidal and barrier-restorative effects, offering a novel therapeutic avenue for combating resistant intestinal infections and associated complications. Moreover, the synergistic strategy employing clove oil also has demonstrated promising therapeutic potential in burn infection models. A study reported that a levofloxacin-loaded clove oil nanoscale emulgel effectively penetrated and disrupted Pseudomonas aeruginosa biofilms, and in a mouse burn wound model, this formulation promoted rapid debridement, enhanced re-epithelialisation and collagen deposition, and accelerated wound healing [48]. These findings corroborate that clove oil not only directly inhibits biofilm formation but also synergises with antibiotics to improve their penetration and efficacy. This supports the potential of clove oil-based combinations as a viable strategy for managing resistant biofilm-associated infections (e.g., in burn wounds or colitis). Given that the clove oil used in this study was standardized to contain over 85% eugenol, the observed inhibition of the QS pathways is likely attributable to this primary phenolic component, which is consistent with previous reports on the anti-virulence properties of eugenol [49,50].

Clove oil was identified as a candidate due to its ability to target the LuxS/AI-2 QS system, and acts as both a biofilm inhibitor and an antibiotic synergist. The synergy is attributed to the ability of clove oil to weaken bacterial defence mechanisms, namely biofilm formation and motility, thereby making the bacteria more susceptible to the bactericidal effects of fosfomycin sodium [51,52]. By delivering a dual benefit of enhanced bacterial clearance and suppression of adaptive stress responses, this combination therapy provides a promising experimental strategy for treating serious *E. coli O157* infections. It should be noted that this study focused exclusively on the reference strain ATCC 43895. While this provided a controlled environment to elucidate the specific mechanisms of and QS inhibition, future research is warranted to evaluate the efficacy of clove oil against a diverse panel of clinical *E. coli* isolates.

## 5. Conclusions

This study demonstrates that clove oil exerts potent anti-virulence activity against *E. coli* O157 by targeting key pathogenic behaviours, specifically through the inhibition of the LuxS/AI-2 QS pathway. We found that clove oil suppresses bacterial motility and disrupts biofilm formation in a concentration-dependent manner, effectively regulating the synthesis and transport of AI-2 by inhibiting *luxS* and QS-related genes (e.g., *lsrA*, *lsrB*, *lsrG*, *lsrK*, and *lsrF*). Crucially, clove oil acts as an effective adjuvant to fosfomycin sodium, exhibiting significant synergistic potential both in vitro and in vivo. In a murine peritoneal infection model, the combination therapy significantly improved survival, reduced systemic bacterial loads in the liver and spleen, and alleviated histopathological damage. While we observed improvements in intestinal barrier integrity in this systemic infection model, these effects are attributed to the reduction of systemic inflammation and bacterial dissemination, rather than a direct probiotic modulation of the gut microbiota. In summary, these findings highlight a novel combinatorial strategy utilizing clove oil via biofilm disruption to potentiate fosfomycin sodium efficacy against refractory infections caused by *E. coli O15*7. Further studies are warranted to validate this strategy against a broader panel of clinical isolates. Notably, given the pivotal role of fosfomycin sodium in clinical urology, this combinatorial approach may offer a viable strategy for managing refractory urinary tract infections. Nevertheless, continued investigation is needed to substantiate its therapeutic potential prior to clinical translation.

## Figures and Tables

**Table 1:** Primer sequences for PCR analysis.

Target Genes		Sequences (5′–3′)
*luxS*	Forward	GGATCCTCTAGAATGCCGTTGTTAGATAGCTTCACAG
	Reverse	TGCTCACCCTCCGATGTGCAGTTCCTGCAACTTCTC
*luxS*-eGFP	Forward	TACGCCAAGCTTATGCCGTTGTTAGATAGCTTCACAG
	Reverse	GGATCCTCTAGATTACTTGTACAGCTCGTCCATGCC

**Table 2:** The primer sequences for qRT-PCR.

Target Genes		Sequences (5′–3′)
*luxS*	Forward	GAAAACAATGAACACCCCGCATGG
	Reverse	TCCCTCTTTCTGGCATCACTTCTTTG
*csgD*	Forward	AATCGCTGGCAATTACAGG
	Reverse	CCGCTTCCATCATATCCAG
*flhC*	Forward	ATGCTGCCATTCTCAACCGACTG
	Reverse	CGCATCGACGCCATTACACAAAC
*flhD*	Forward	CGTTAGCGGCACTGACTCTTCC
	Reverse	TTGCGTCAACTGAGTAATCGTCTGG
*lsrA*	Forward	GATGAACCTACCGCCTCGCTTAC
	Reverse	AACAATACCCACGCCAGTAGCAAG
*lsrB*	Forward	AGTGCTGACCTGGGACTCTGATAC
	Reverse	GCCATATCCACCAACATACCTCCTAAC
*lsrF*	Forward	TCGGCAGCGAATATGAACATCAGTC
	Reverse	CCATATCTTTGCCCACGCCAGTC
*lsrK*	Forward	GATGAACCTACCGCCTCGCTTAC
	Reverse	AACAATACCCACGCCAGTAGCAAG
*fliC*	Forward	TTACCAACCTGAACAACACCACTACC
	Reverse	ACATATTGGACACTTCGGTCGCATAG
*fliM*	Forward	ATGGGCGATAGTATTCTTTCTCAAGCT
	Reverse	TCATTTGGGCTGTTCCTCGT
*flin*	Forward	CAATGGACGATCTGTGGGCTGAAG
	Reverse	CACCGCCAAATTGCTGGAACAC

## Data Availability

The original contributions presented in this study are included in the article. Further inquiries can be directed to the corresponding authors.
